# 4-[(5-Bromo-2-hy­droxy­benzyl­idene)amino]-3-ethyl-1*H*-1,2,4-triazole-5(4*H*)-thione

**DOI:** 10.1107/S1600536814016833

**Published:** 2014-07-26

**Authors:** Cai-Xia Yuan, Shu-Fen Lan, Xin-Yu Liu, Miao-Li Zhu

**Affiliations:** a Institute of Molecular Science, Key Laboratory of Chemical Biology and Molecular, Engineering of the Education Ministry, Shanxi University, Taiyuan, Shanxi 030006, People’s Republic of China

**Keywords:** crystal structure

## Abstract

The title compound, C_11_H_11_BrN_4_OS, crystallized as a racemic twin with two symmetry-independent mol­ecules in the asymmetric unit. The dihedral angles between the benzene and triazole rings of the two independent mol­ecules are 56.41 (18) and 54.48 (18)°. An intra­molecular O—H⋯N hydrogen bond occurs in each mol­ecule. In the crystal, pairs of symmetry-independent mol­ecules are linked by pairs of almost linear N—H⋯S hydrogen bonds, forming cyclic dimers characterized by an *R*_2_^2^(8) motif. There are weak π–π inter­actions between the benzene rings of symmetry-independent mol­ecules, with a centroid–centroid distance of 3.874 (3) Å.

## Related literature   

For background to the biological activity of related compounds, see: Demirbas (2004 ▶); Demirbas *et al.* (2009 ▶); Todoulou *et al.* (1994 ▶); Kumar *et al.* (2008 ▶); Kochikyan *et al.* (2011 ▶); Singhal *et al.* (2011 ▶); Popiołek *et al.* (2013 ▶); Sraa (2012 ▶). For similar structures, see: Wu *et al.* (2012 ▶); Pannu & Hundal (2011 ▶). For standard bond lengths, see: Allen *et al.* (1987 ▶). For graph-sets of hydrogen-bond motifs, see: Bernstein *et al.* (1995 ▶).
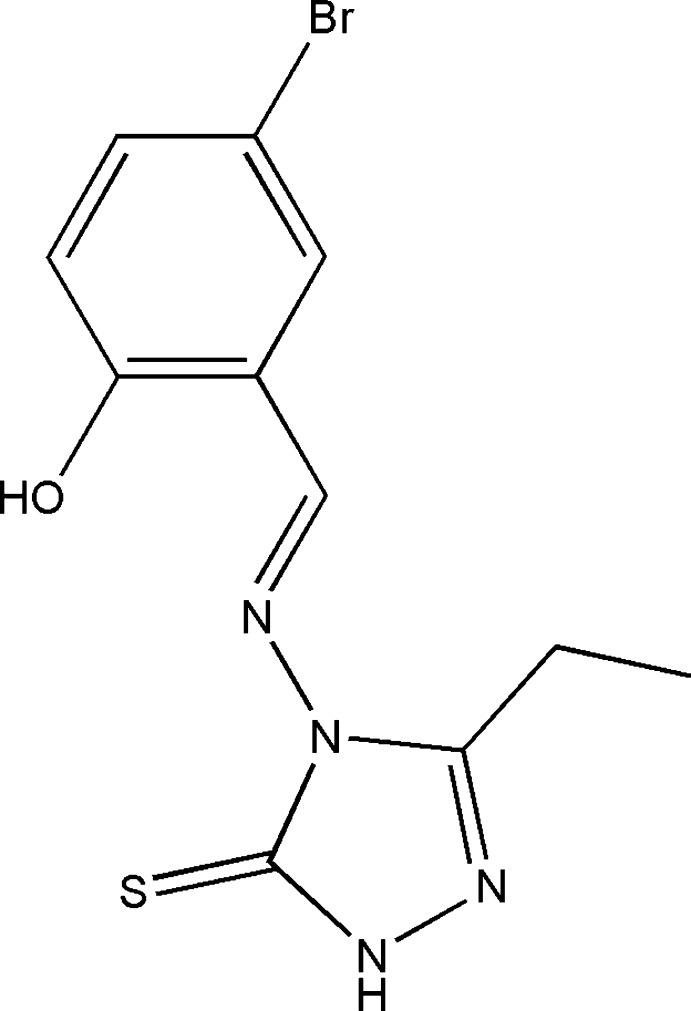


## Experimental   

### 

#### Crystal data  

C_11_H_11_BrN_4_OS
*M* *_r_* = 327.21
Monoclinic, 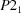

*a* = 6.323 (4) Å
*b* = 16.459 (11) Å
*c* = 12.461 (8) Å
β = 90.330 (9)°
*V* = 1296.8 (15) Å^3^
*Z* = 4
Mo *K*α radiation
μ = 3.32 mm^−1^
*T* = 298 K
0.40 × 0.35 × 0.30 mm

#### Data collection  

Bruker SMART APEXII diffractometer
Absorption correction: multi-scan (*SADABS*; Sheldrick, 2000 ▶) *T*_min_ = 0.350, *T*_max_ = 0.435
14802 measured reflections
5222 independent reflections
4285 reflections with *I* > 2σ(*I*)
*R*_int_ = 0.033

#### Refinement  

*R*[*F* ^2^ > 2σ(*F* ^2^)] = 0.031
*wR*(*F* ^2^) = 0.065
*S* = 1.01
5222 reflections
328 parameters
1 restraint
H-atom parameters constrained
Δρ_max_ = 0.28 e Å^−3^
Δρ_min_ = −0.45 e Å^−3^
Absolute structure: Flack (1983 ▶), 2514 Friedel pairs
Absolute structure parameter: 0.581 (7)

### 

Data collection: *SMART* (Bruker, 2000 ▶); cell refinement: *SAINT* (Bruker, 2000 ▶); data reduction: *SAINT*; program(s) used to solve structure: *SHELXS97* (Sheldrick, 2008 ▶); program(s) used to refine structure: *SHELXL97* (Sheldrick, 2008 ▶); molecular graphics: *SHELXTL/PC* (Sheldrick, 2008 ▶); software used to prepare material for publication: *SHELXTL/PC*.

## Supplementary Material

Crystal structure: contains datablock(s) I. DOI: 10.1107/S1600536814016833/fy2115sup1.cif

Structure factors: contains datablock(s) I. DOI: 10.1107/S1600536814016833/fy2115Isup2.hkl

Supporting information file. DOI: 10.1107/S1600536814016833/fy2115Isup3.cml

CCDC reference: 1015277

Additional supporting information:  crystallographic information; 3D view; checkCIF report

## Figures and Tables

**Table 1:** Hydrogen-bond geometry (Å, °)

*D*—H⋯*A*	*D*—H	H⋯*A*	*D*⋯*A*	*D*—H⋯*A*
N1—H1⋯S2^i^	0.86	2.45	3.309 (3)	176
N5—H5*A*⋯S1^ii^	0.86	2.44	3.302 (3)	177
O1—H1*A*⋯N4	0.82	2.02	2.712 (4)	141
O2—H2⋯N8	0.82	1.99	2.695 (4)	143

Symmetry codes: (i) 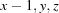; (ii) 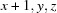.

**Fig. 1.**
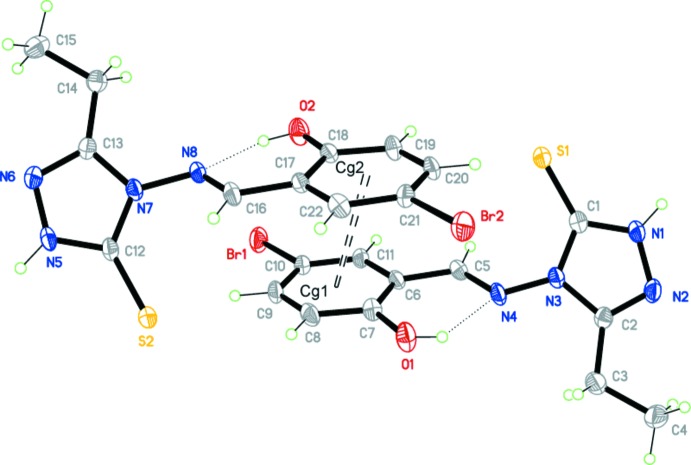
View of the structure with displacement ellipsoids drawn at the 30% probability level. Dotted lines represent hydrogen bonds and π–π interactions.

**Fig. 2.**
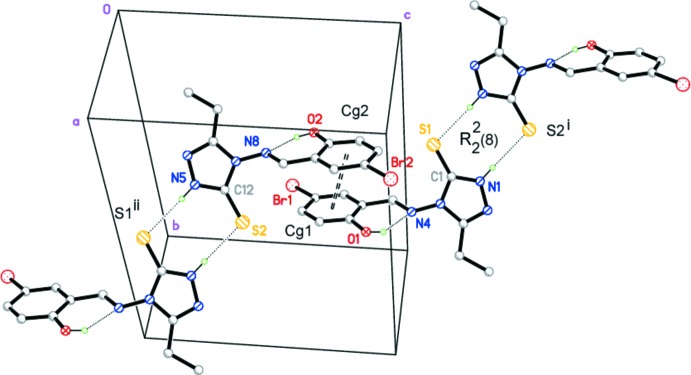
A part of the crystal structure, showing the formation of a chain of R22(8) hydrogen-bonded rings and π-π stacking between the benzene rings rings; Cg1: C6/C7/C8/C9/C10/C11, Cg2: C17/C18/C19/C20/C21/C22. Symmetry codes: i) x - 1, y. z + 1; ii) x + 1, y, z - 1. H atoms without H-bonds have been omitted for clarity.

## supplementary crystallographic information

### S1. Comment

Recently, 1,2,4-triazoles and their derivatives have been the focus of a great deal of attention owing to their effective biological activities such as antimicrobial, antiviral, analgesic, anti-inflammatory, anticancer and antioxidant properties (Demirbas *et al.*, 2004 and 2009; Kochikyan *et al.*, 2011; Kumar *et al.*, 2008; Singhal *et al.*, 2011; Todoulou *et al.*, 1994). As a result, a number of attempts were made to improve the activity of these compounds by varying the substituents on the 1,2,4-triazole nucleus (Popiołek *et al.*, 2013; Sraa *et al.*, 2012). Among these, the amino- and mercapto-group substituted 1,2,4-triazole ring systems represent an important group of compounds that are promising for practical application. Therefore, the title compound (I), has been synthesized and its crystal structure has been determinined.

The crystal structure is illustrated in Fig. 1. The title compound (I) crystallizes in the monoclinic space group *P*2_1_ with two symmetry-independent molecules in the unit cell. The bond lengths of N4–C5 [1.274 (5) Å] and N8–C16 [1.272 (5) Å] confirm them as double bonds, which is similar to those reported in other Schiff bases (Pannu *et al.*, 2011; Wu *et al.*, 2012;). The molecule of (I) exists in the thione tautometic form, with C═S distances of 1.673 (4) and 1.672 (4) Å, which indicates a substantial double-bond character (Allen *et al.*, 1987).

The packing arrangement in the crystal structure of (I) is shown in Fig. 2. As a common feature of *o*-hydroxysalicylidene systems, the azomethine group in title compound forms intramolecular O–H···N hydrogen bonds with the neighbouring hydroxyl groups. Moreover, the crystal structure also contains intermolecular N–H···S hydrogen bonds between both independent molecules with cyclic motifs [graph set *R*_2_^2^(8)] (Bernstein *et al.*, 1995). The molecules are further linked *via* weak π-π interactions between benzene rings (*Cg*1 and *Cg*2). The hydrogen bonds and π-π interactions link the molecules into ribbon structures.

### S2. Experimental

The title compound was synthesized by condensation of 4-amino-3-ethyl- 1*H*-1,2,4-triazole-5(4*H*)-thione and 5-Br-salicylaldehyde. 0.5 mmol of 4-amino-3-ethyl-1,2,4-triazole-5-thione was thoroughly dissolved in 20 ml of ethanol with a constant stirring at 353 K. Then 0.5 mmol of 5-bromosalicylaldehyde in 10 ml ethanol was added dropwise to a solution of the above. The mixture was further refluxed for 2 h. The resulting yellow solution was filtered and the filtrate was left to stand at room temperature. The yellow crystals of compound (I) were received from the filtrate with slowly evaporating the solvent for a few days. Yield: 78%. Anal. Calcd. for C_11_H_11_BrN_4_OS: C 40.38, H 3.39, N 17.12%. Found: C 40.31, H 3.45, N 17.07%. IR (ν/cm^-1^): 3109, 3055, 2958, 1603, 1588, 1513, 1416, 1352, 1288, 1165, 1174, 967, 817, 627. UV/vis in DMSO, λ_max_/nm (ε 10^3^/*M*^-1^ cm^-1^): 265(13.9), 343(7.77).

### S3. Refinement

The H atoms bonded to C atoms were placed in calculated positions (C—H=0.96, 0.97 and 0.93 Å for C*sp*^3^, C*sp*^2^ and C*sp* atoms, respectively), assigned fixed *U*_iso_ values [*U*_iso_(H) = 1.5 *U*_eq_(C) for methyl groups and 1.2 *U*_eq_(C) for all others] and treated as riding atoms. The H atoms attached to O and N atoms were found in difference electron-density maps and were refined isotropically, with *U*_iso_(H) = 1.5 *U*_eq_(O) or *U*_iso_(H) = 1.2 *U*_eq_(N) and fixed O—H (0.82 Å) and N—H (0.86 Å) bond lengths.

### Crystal data

**Table:**

C_11_H_11_BrN_4_OS	*F*(000) = 656
*M**_r_* = 327.21	*D*_x_ = 1.676 Mg m^−^^3^
Monoclinic, *P*2_1_	Mo *K*α radiation, λ = 0.71073 Å
Hall symbol: P 2yb	Cell parameters from 4187 reflections
*a* = 6.323 (4) Å	θ = 2.5–25.3°
*b* = 16.459 (11) Å	µ = 3.32 mm^−^^1^
*c* = 12.461 (8) Å	*T* = 298 K
β = 90.330 (9)°	Block, yellow
*V* = 1296.8 (15) Å^3^	0.40 × 0.35 × 0.30 mm
*Z* = 4	

### Data collection

**Table:**

Bruker SMART APEXII diffractometer	5222 independent reflections
Radiation source: fine-focus sealed tube	4285 reflections with *I* > 2σ(*I*)
Graphite monochromator	*R*_int_ = 0.033
ω scans	θ_max_ = 26.3°, θ_min_ = 1.6°
Absorption correction: multi-scan (*SADABS*; Sheldrick, 2000)	*h* = −7→7
*T*_min_ = 0.350, *T*_max_ = 0.435	*k* = −20→20
14802 measured reflections	*l* = −15→15

### Refinement

**Table:**

Refinement on *F*^2^	Secondary atom site location: difference Fourier map
Least-squares matrix: full	Hydrogen site location: inferred from neighbouring sites
*R*[*F*^2^ > 2σ(*F*^2^)] = 0.031	H-atom parameters constrained
*wR*(*F*^2^) = 0.065	*w* = 1/[σ^2^(*F*_o_^2^) + (0.031*P*)^2^] where *P* = (*F*_o_^2^ + 2*F*_c_^2^)/3
*S* = 1.01	(Δ/σ)_max_ = 0.001
5222 reflections	Δρ_max_ = 0.28 e Å^−^^3^
328 parameters	Δρ_min_ = −0.45 e Å^−^^3^
1 restraint	Absolute structure: Flack (1983), 2514 Friedel pairs
Primary atom site location: structure-invariant direct methods	Absolute structure parameter: 0.581 (7)

### Special details

**Table:**

Geometry. All e.s.d.'s (except the e.s.d. in the dihedral angle between two l.s. planes) are estimated using the full covariance matrix. The cell e.s.d.'s are taken into account individually in the estimation of e.s.d.'s in distances, angles and torsion angles; correlations between e.s.d.'s in cell parameters are only used when they are defined by crystal symmetry. An approximate (isotropic) treatment of cell e.s.d.'s is used for estimating e.s.d.'s involving l.s. planes.
Refinement. Refinement of *F*^2^ against ALL reflections. The weighted *R*-factor *wR* and goodness of fit *S* are based on *F*^2^, conventional *R*-factors *R* are based on *F*, with *F* set to zero for negative *F*^2^. The threshold expression of *F*^2^ > σ(*F*^2^) is used only for calculating *R*-factors(gt) *etc*. and is not relevant to the choice of reflections for refinement. *R*-factors based on *F*^2^ are statistically about twice as large as those based on *F*, and *R*- factors based on ALL data will be even larger.

### Fractional atomic coordinates and isotropic or equivalent isotropic displacement parameters (Å^2^)

**Table:**

	*x*	*y*	*z*	*U*_iso_*/*U*_eq_	
Br1	0.19835 (6)	0.61341 (2)	−0.42475 (3)	0.05443 (12)	
S1	0.10279 (14)	0.43885 (6)	0.11498 (7)	0.0394 (2)	
O1	0.8327 (4)	0.49113 (17)	−0.10062 (19)	0.0511 (7)	
H1A	0.8001	0.5035	−0.0391	0.077*	
N1	0.2852 (5)	0.50342 (19)	0.2940 (2)	0.0425 (8)	
H1	0.1906	0.4848	0.3368	0.051*	
N2	0.4568 (5)	0.5484 (2)	0.3291 (2)	0.0423 (8)	
N3	0.4567 (4)	0.53213 (17)	0.1540 (2)	0.0313 (7)	
N4	0.5457 (4)	0.53192 (18)	0.0511 (2)	0.0360 (7)	
C1	0.2787 (5)	0.4913 (2)	0.1880 (3)	0.0325 (8)	
C2	0.5590 (5)	0.5653 (2)	0.2428 (3)	0.0337 (8)	
C3	0.7562 (6)	0.6128 (3)	0.2360 (3)	0.0438 (9)	
H3A	0.7331	0.6594	0.1898	0.053*	
H3B	0.8655	0.5795	0.2039	0.053*	
C4	0.8317 (7)	0.6420 (2)	0.3461 (3)	0.0529 (11)	
H4A	0.7232	0.6744	0.3786	0.079*	
H4B	0.9574	0.6741	0.3380	0.079*	
H4C	0.8619	0.5960	0.3909	0.079*	
C5	0.4165 (6)	0.5478 (2)	−0.0249 (3)	0.0332 (8)	
H5	0.2777	0.5616	−0.0087	0.040*	
C6	0.4824 (5)	0.5449 (2)	−0.1370 (3)	0.0305 (8)	
C7	0.6786 (5)	0.5157 (2)	−0.1695 (3)	0.0343 (8)	
C8	0.7249 (6)	0.5113 (2)	−0.2784 (3)	0.0404 (10)	
H8	0.8531	0.4893	−0.3004	0.048*	
C9	0.5816 (6)	0.5393 (2)	−0.3542 (3)	0.0390 (9)	
H9	0.6142	0.5370	−0.4268	0.047*	
C10	0.3887 (6)	0.5707 (2)	−0.3211 (3)	0.0362 (9)	
C11	0.3376 (6)	0.5723 (2)	−0.2137 (3)	0.0342 (8)	
H11	0.2063	0.5917	−0.1923	0.041*	
Br2	0.85545 (7)	0.24611 (3)	0.99904 (3)	0.06106 (14)	
S2	0.93822 (14)	0.42617 (6)	0.46355 (7)	0.0375 (2)	
O2	0.2087 (4)	0.36500 (17)	0.6796 (2)	0.0480 (8)	
H2	0.2556	0.3672	0.6184	0.072*	
N5	0.7520 (5)	0.36383 (19)	0.2834 (2)	0.0390 (8)	
H5A	0.8466	0.3827	0.2409	0.047*	
N6	0.5811 (5)	0.3204 (2)	0.2478 (2)	0.0401 (8)	
N7	0.5821 (4)	0.33383 (18)	0.4232 (2)	0.0314 (7)	
N8	0.4955 (4)	0.33164 (19)	0.5266 (2)	0.0321 (7)	
C12	0.7603 (5)	0.3746 (2)	0.3909 (3)	0.0319 (8)	
C13	0.4769 (5)	0.3032 (2)	0.3348 (3)	0.0315 (8)	
C14	0.2795 (5)	0.2556 (2)	0.3413 (3)	0.0400 (8)	
H14A	0.1713	0.2884	0.3750	0.048*	
H14B	0.3037	0.2083	0.3863	0.048*	
C15	0.1996 (6)	0.2275 (2)	0.2317 (3)	0.0472 (10)	
H15A	0.1887	0.2735	0.1845	0.071*	
H15B	0.0630	0.2028	0.2393	0.071*	
H15C	0.2967	0.1887	0.2023	0.071*	
C16	0.6252 (6)	0.3124 (2)	0.6007 (3)	0.0353 (9)	
H16	0.7633	0.2985	0.5832	0.042*	
C17	0.5601 (6)	0.3117 (2)	0.7123 (3)	0.0328 (8)	
C18	0.3609 (6)	0.3392 (2)	0.7470 (3)	0.0361 (9)	
C19	0.3173 (6)	0.3417 (2)	0.8567 (3)	0.0443 (9)	
H19	0.1881	0.3620	0.8797	0.053*	
C20	0.4612 (6)	0.3147 (2)	0.9307 (3)	0.0462 (10)	
H20	0.4296	0.3165	1.0034	0.055*	
C21	0.6543 (6)	0.2846 (2)	0.8971 (3)	0.0423 (9)	
C22	0.7078 (6)	0.2850 (2)	0.7897 (3)	0.0403 (9)	
H22	0.8411	0.2675	0.7685	0.048*	

### Atomic displacement parameters (Å^2^)

**Table:**

	*U*^11^	*U*^22^	*U*^33^	*U*^12^	*U*^13^	*U*^23^
Br1	0.0537 (2)	0.0799 (3)	0.02963 (19)	0.0011 (2)	−0.00409 (16)	0.0045 (2)
S1	0.0403 (5)	0.0497 (6)	0.0281 (4)	−0.0051 (4)	0.0038 (4)	0.0017 (4)
O1	0.0457 (16)	0.074 (2)	0.0334 (14)	0.0183 (14)	0.0076 (12)	0.0048 (14)
N1	0.0402 (18)	0.064 (2)	0.0238 (16)	−0.0095 (16)	0.0092 (13)	0.0011 (15)
N2	0.0442 (18)	0.058 (2)	0.0251 (15)	−0.0021 (16)	0.0042 (14)	−0.0043 (14)
N3	0.0337 (16)	0.0378 (18)	0.0226 (15)	0.0022 (14)	0.0057 (12)	0.0023 (13)
N4	0.0364 (17)	0.047 (2)	0.0252 (16)	0.0010 (14)	0.0102 (13)	0.0031 (14)
C1	0.034 (2)	0.036 (2)	0.0277 (19)	0.0065 (16)	0.0038 (15)	0.0053 (15)
C2	0.035 (2)	0.036 (2)	0.0300 (19)	0.0093 (16)	0.0040 (15)	−0.0019 (15)
C3	0.047 (2)	0.044 (2)	0.041 (2)	−0.002 (2)	0.0050 (16)	0.003 (2)
C4	0.062 (3)	0.047 (3)	0.049 (2)	−0.013 (2)	−0.008 (2)	0.0012 (19)
C5	0.0341 (19)	0.033 (2)	0.033 (2)	0.0020 (16)	0.0075 (16)	0.0027 (16)
C6	0.0342 (19)	0.031 (2)	0.0260 (18)	−0.0051 (16)	0.0071 (15)	0.0024 (15)
C7	0.036 (2)	0.035 (2)	0.0315 (19)	0.0044 (16)	0.0048 (16)	0.0009 (15)
C8	0.043 (2)	0.043 (2)	0.035 (2)	0.0007 (18)	0.0164 (19)	−0.0040 (17)
C9	0.048 (2)	0.047 (2)	0.0228 (18)	−0.0021 (19)	0.0103 (16)	−0.0042 (16)
C10	0.043 (2)	0.040 (2)	0.0249 (18)	−0.0066 (17)	0.0016 (16)	0.0001 (16)
C11	0.0327 (19)	0.043 (2)	0.0275 (19)	−0.0011 (16)	0.0046 (15)	−0.0031 (16)
Br2	0.0659 (3)	0.0819 (3)	0.0352 (2)	−0.0148 (2)	−0.01050 (19)	0.0139 (2)
S2	0.0369 (5)	0.0487 (6)	0.0268 (4)	−0.0048 (4)	0.0045 (4)	−0.0016 (4)
O2	0.0395 (16)	0.067 (2)	0.0380 (16)	0.0097 (14)	0.0065 (12)	0.0014 (14)
N5	0.0415 (19)	0.053 (2)	0.0224 (16)	−0.0015 (15)	0.0075 (13)	0.0009 (14)
N6	0.0423 (19)	0.055 (2)	0.0233 (16)	−0.0035 (16)	0.0007 (14)	−0.0044 (14)
N7	0.0297 (16)	0.0400 (18)	0.0248 (15)	0.0023 (14)	0.0068 (12)	−0.0010 (13)
N8	0.0317 (16)	0.0435 (19)	0.0213 (16)	−0.0013 (14)	0.0081 (13)	−0.0012 (13)
C12	0.0308 (19)	0.042 (2)	0.0233 (17)	0.0038 (16)	0.0039 (14)	0.0010 (15)
C13	0.034 (2)	0.037 (2)	0.0237 (18)	0.0016 (16)	−0.0003 (15)	−0.0048 (15)
C14	0.0397 (19)	0.043 (2)	0.0373 (19)	−0.0010 (18)	0.0062 (15)	−0.0065 (17)
C15	0.054 (2)	0.041 (3)	0.047 (2)	−0.0062 (19)	−0.0110 (19)	−0.0010 (19)
C16	0.042 (2)	0.038 (2)	0.0264 (19)	0.0009 (17)	0.0117 (17)	0.0011 (16)
C17	0.039 (2)	0.034 (2)	0.0251 (18)	−0.0053 (16)	0.0059 (15)	0.0012 (15)
C18	0.043 (2)	0.033 (2)	0.032 (2)	−0.0094 (17)	0.0074 (17)	−0.0015 (16)
C19	0.049 (2)	0.052 (2)	0.032 (2)	−0.0018 (19)	0.0139 (18)	−0.0057 (18)
C20	0.059 (3)	0.053 (3)	0.0261 (19)	−0.015 (2)	0.0159 (18)	−0.0049 (17)
C21	0.053 (2)	0.049 (2)	0.0247 (18)	−0.0162 (19)	−0.0019 (17)	0.0030 (16)
C22	0.041 (2)	0.043 (2)	0.037 (2)	0.0005 (18)	0.0072 (17)	0.0034 (17)

### Geometric parameters (Å, º)

**Table:**

Br1—C10	1.896 (4)	Br2—C21	1.901 (4)
S1—C1	1.673 (4)	S2—C12	1.672 (4)
O1—C7	1.356 (4)	O2—C18	1.343 (4)
O1—H1A	0.8200	O2—H2	0.8200
N1—C1	1.336 (4)	N5—C12	1.352 (4)
N1—N2	1.383 (4)	N5—N6	1.367 (4)
N1—H1	0.8600	N5—H5A	0.8600
N2—C2	1.288 (4)	N6—C13	1.303 (4)
N3—C1	1.380 (4)	N7—C12	1.374 (4)
N3—C2	1.391 (4)	N7—C13	1.379 (4)
N3—N4	1.403 (4)	N7—N8	1.404 (4)
N4—C5	1.275 (4)	N8—C16	1.271 (4)
C2—C3	1.474 (5)	C13—C14	1.476 (5)
C3—C4	1.527 (5)	C14—C15	1.525 (5)
C3—H3A	0.9700	C14—H14A	0.9700
C3—H3B	0.9700	C14—H14B	0.9700
C4—H4A	0.9600	C15—H15A	0.9600
C4—H4B	0.9600	C15—H15B	0.9600
C4—H4C	0.9600	C15—H15C	0.9600
C5—C6	1.461 (5)	C16—C17	1.453 (5)
C5—H5	0.9300	C16—H16	0.9300
C6—C7	1.393 (5)	C17—C18	1.408 (5)
C6—C11	1.394 (5)	C17—C22	1.409 (5)
C7—C8	1.392 (5)	C18—C19	1.396 (5)
C8—C9	1.384 (5)	C19—C20	1.366 (5)
C8—H8	0.9300	C19—H19	0.9300
C9—C10	1.390 (5)	C20—C21	1.384 (5)
C9—H9	0.9300	C20—H20	0.9300
C10—C11	1.379 (5)	C21—C22	1.382 (5)
C11—H11	0.9300	C22—H22	0.9300
			
C7—O1—H1A	109.5	C18—O2—H2	109.5
C1—N1—N2	114.3 (3)	C12—N5—N6	114.6 (3)
C1—N1—H1	122.8	C12—N5—H5A	122.7
N2—N1—H1	122.8	N6—N5—H5A	122.7
C2—N2—N1	104.4 (3)	C13—N6—N5	104.3 (3)
C1—N3—C2	108.9 (3)	C12—N7—C13	109.7 (3)
C1—N3—N4	127.7 (3)	C12—N7—N8	127.4 (3)
C2—N3—N4	122.8 (3)	C13—N7—N8	122.3 (3)
C5—N4—N3	114.8 (3)	C16—N8—N7	114.8 (3)
N1—C1—N3	102.2 (3)	N5—C12—N7	101.5 (3)
N1—C1—S1	129.1 (3)	N5—C12—S2	128.7 (3)
N3—C1—S1	128.7 (3)	N7—C12—S2	129.8 (3)
N2—C2—N3	110.2 (3)	N6—C13—N7	109.9 (3)
N2—C2—C3	126.3 (3)	N6—C13—C14	126.4 (3)
N3—C2—C3	123.5 (3)	N7—C13—C14	123.7 (3)
C2—C3—C4	112.1 (3)	C13—C14—C15	112.8 (3)
C2—C3—H3A	109.2	C13—C14—H14A	109.0
C4—C3—H3A	109.2	C15—C14—H14A	109.0
C2—C3—H3B	109.2	C13—C14—H14B	109.0
C4—C3—H3B	109.2	C15—C14—H14B	109.0
H3A—C3—H3B	107.9	H14A—C14—H14B	107.8
C3—C4—H4A	109.5	C14—C15—H15A	109.5
C3—C4—H4B	109.5	C14—C15—H15B	109.5
H4A—C4—H4B	109.5	H15A—C15—H15B	109.5
C3—C4—H4C	109.5	C14—C15—H15C	109.5
H4A—C4—H4C	109.5	H15A—C15—H15C	109.5
H4B—C4—H4C	109.5	H15B—C15—H15C	109.5
N4—C5—C6	121.3 (3)	N8—C16—C17	120.8 (3)
N4—C5—H5	119.4	N8—C16—H16	119.6
C6—C5—H5	119.4	C17—C16—H16	119.6
C7—C6—C11	119.7 (3)	C18—C17—C22	118.7 (3)
C7—C6—C5	123.2 (3)	C18—C17—C16	123.3 (3)
C11—C6—C5	117.0 (3)	C22—C17—C16	117.9 (3)
O1—C7—C8	116.6 (3)	O2—C18—C19	117.3 (3)
O1—C7—C6	123.9 (3)	O2—C18—C17	123.3 (3)
C8—C7—C6	119.6 (3)	C19—C18—C17	119.4 (4)
C9—C8—C7	120.5 (3)	C20—C19—C18	121.1 (4)
C9—C8—H8	119.7	C20—C19—H19	119.4
C7—C8—H8	119.7	C18—C19—H19	119.4
C8—C9—C10	119.6 (3)	C19—C20—C21	119.8 (3)
C8—C9—H9	120.2	C19—C20—H20	120.1
C10—C9—H9	120.2	C21—C20—H20	120.1
C11—C10—C9	120.4 (3)	C22—C21—C20	120.8 (4)
C11—C10—Br1	120.2 (3)	C22—C21—Br2	118.8 (3)
C9—C10—Br1	119.4 (3)	C20—C21—Br2	120.3 (3)
C10—C11—C6	120.1 (3)	C21—C22—C17	119.9 (3)
C10—C11—H11	119.9	C21—C22—H22	120.0
C6—C11—H11	119.9	C17—C22—H22	120.0
			
C1—N1—N2—C2	0.5 (4)	C12—N5—N6—C13	0.2 (4)
C1—N3—N4—C5	51.3 (5)	C12—N7—N8—C16	−51.8 (5)
C2—N3—N4—C5	−139.1 (4)	C13—N7—N8—C16	138.0 (4)
N2—N1—C1—N3	−0.7 (4)	N6—N5—C12—N7	0.7 (4)
N2—N1—C1—S1	178.4 (3)	N6—N5—C12—S2	−177.9 (3)
C2—N3—C1—N1	0.6 (4)	C13—N7—C12—N5	−1.3 (4)
N4—N3—C1—N1	171.4 (3)	N8—N7—C12—N5	−172.5 (3)
C2—N3—C1—S1	−178.5 (3)	C13—N7—C12—S2	177.3 (3)
N4—N3—C1—S1	−7.7 (5)	N8—N7—C12—S2	6.1 (5)
N1—N2—C2—N3	−0.1 (4)	N5—N6—C13—N7	−1.0 (4)
N1—N2—C2—C3	179.9 (3)	N5—N6—C13—C14	−179.0 (3)
C1—N3—C2—N2	−0.4 (4)	C12—N7—C13—N6	1.6 (4)
N4—N3—C2—N2	−171.7 (3)	N8—N7—C13—N6	173.3 (3)
C1—N3—C2—C3	179.7 (3)	C12—N7—C13—C14	179.6 (3)
N4—N3—C2—C3	8.3 (5)	N8—N7—C13—C14	−8.7 (5)
N2—C2—C3—C4	−0.3 (6)	N6—C13—C14—C15	−1.0 (6)
N3—C2—C3—C4	179.6 (3)	N7—C13—C14—C15	−178.7 (3)
N3—N4—C5—C6	−176.1 (3)	N7—N8—C16—C17	176.8 (3)
N4—C5—C6—C7	7.9 (6)	N8—C16—C17—C18	−6.3 (6)
N4—C5—C6—C11	−172.8 (3)	N8—C16—C17—C22	176.1 (3)
C11—C6—C7—O1	177.0 (3)	C22—C17—C18—O2	−179.2 (3)
C5—C6—C7—O1	−3.7 (6)	C16—C17—C18—O2	3.3 (6)
C11—C6—C7—C8	−2.4 (5)	C22—C17—C18—C19	2.0 (6)
C5—C6—C7—C8	176.9 (3)	C16—C17—C18—C19	−175.6 (4)
O1—C7—C8—C9	−176.4 (3)	O2—C18—C19—C20	178.4 (4)
C6—C7—C8—C9	3.1 (6)	C17—C18—C19—C20	−2.8 (6)
C7—C8—C9—C10	−1.1 (6)	C18—C19—C20—C21	0.3 (6)
C8—C9—C10—C11	−1.6 (6)	C19—C20—C21—C22	3.0 (6)
C8—C9—C10—Br1	177.2 (3)	C19—C20—C21—Br2	−179.5 (3)
C9—C10—C11—C6	2.2 (6)	C20—C21—C22—C17	−3.7 (6)
Br1—C10—C11—C6	−176.5 (3)	Br2—C21—C22—C17	178.8 (3)
C7—C6—C11—C10	−0.2 (5)	C18—C17—C22—C21	1.2 (5)
C5—C6—C11—C10	−179.5 (3)	C16—C17—C22—C21	178.9 (4)

### Hydrogen-bond geometry (Å, º)

**Table:**

*D*—H···*A*	*D*—H	H···*A*	*D*···*A*	*D*—H···*A*
N1—H1···S2^i^	0.86	2.45	3.309 (3)	176
N5—H5*A*···S1^ii^	0.86	2.44	3.302 (3)	177
O1—H1*A*···N4	0.82	2.02	2.712 (4)	141
O2—H2···N8	0.82	1.99	2.695 (4)	143

Symmetry codes: (i) *x*−1, *y*, *z*; (ii) *x*+1, *y*, *z*.
